# Editorial: Radiomics in prostate cancer imaging

**DOI:** 10.3389/fonc.2022.1010901

**Published:** 2022-09-23

**Authors:** Luca Brunese, Pasquale Martino, Massimo Mischi, Mukesh Prasad, Antonella Santone

**Affiliations:** ^1^ Department of Medicine and Health Sciences “Vincenzo Tiberio”, University of Molise, Campobasso, Italy; ^2^ Azienda Ospedaliero Universitaria Consorziale Policlinico di Bari, Bari, Italy; ^3^ Eindhoven University of Technology, Eindhoven, Netherlands; ^4^ University of Technology Sydney, Sydney, NSW, Australia

**Keywords:** radiomics, imaging, artificial intelligence, prostate, cancer

Prostate cancer originates from the cells inside the prostate gland, which begin to grow out of control. About 1 in men are diagnosed with prostate cancer in their life-time. In US, for instance, prostate cancer is the most common cancer in the male population and accounts for 27% percent of all cancers diagnosed in men: in 2022, an estimated 268,490 new cases of prostate cancer will be diagnosed in the US and 34,500 men will die from the disease. Despite the high incidence, prostate cancer death rates declined by about half from the mid-1990s to the mid-2010s due to earlier detection through Prostate Specific Antigen (PSA) testing and advances in treatment. However, decline has slowed in recent years, likely reflecting the uptick in distant-stage diagnoses; from 2015 to 2019, the rate decreased by 0.6% per year^1^. This is also demonstrated by the data on the five-year survival after diagnosis - on average 92 percent - a percentage among the highest for cancer disease, especially if we take into account the advanced average age of the patients.

Prostate cancer is suspected either by measuring PSA in the blood or by digital rectal examination (DRE) of the gland. Once a tumour is suspected, the diagnosis is made with a systematic prostate biopsy, an invasive and uncomfortable examination for the patient, with associated risks of developing infections. More recently, however, prebiopsy multiparametric MRI is advised by the guidelines of the European Association of Urology.

As a matter of fact, histological analysis of biopsy specimens is able to determine with certainty the presence of cancer cells in the prostate tissue. The systematic biopsy procedure consists of taking small samples from different areas of the prostate gland according to a predefined grid. The samples are most commonly taken transrectally under transrectal ultrasound guidance, although transperineal biopsies are gaining attention because of the reduced risk of infections. A positive result confirms the presence of prostate cancer. A pathologist assigns a Gleason score to cancer cells found in the biopsy specimen based on their microscopic appearance. Because of the limited spatial sampling (typically 12 biopsy cores), a negative biopsy session cannot exclude with 100% certainty the presence of cancer cells. Therefore, the patient will enter a surveillance phase with further periodic checks.

Recent radiological techniques such as CT, Magnetic Resonance, ultrasound and PET are able to generate images of the highest quality that in recent years have contributed to improving the diagnosis and monitoring of various pathologies, especially oncological ones. Recently, the results of these tests have begun to be viewed not only as images, but also as valuable data resources available to the scientific community in order to improve the human knowledge in fighting pathologies.

In fact, in recent years the research direction consists of using medical images to identify information that might otherwise be overlooked by the human eye. In this context, radiomics is establishing itself as a new discipline that deals with extrapolating many features from radiological images which are then processed through appropriate data analysis methods. This method can allow more accurate diagnoses, but it can also provide valuable information on specific tumour characteristics, such as predicting the response to a treatment, predicting metastasis or highlighting the presence of particular genetic and epigenetic alterations, which would otherwise be obtainable only by using invasive procedures.

As a new milestone in diagnostic imaging, radiomic analysis applied in the oncology field can allow providing valuable information on the characteristics of tumours, on any mutations and on the personalization of oncological therapies.

The aim of radiomics applied in prostate cancer imaging is to boost the development of novel methodologies and techniques to increase the amount of information (qualitative and quantitative) that can be extracted from medical images, by providing a way to analyse this information automatically. The ultimate goal is the use of radiomics for timely diagnosis of prostate cancer, possibly enabling personalized therapy based on accurate prognostic information. Further long-term goals include the prediction of the risk of developing prostate cancer. These aspects are clearly reflected in the research papers accepted in the Radiomics in Prostate Cancer Imaging Research Topic: for instance, multiparametric magnetic resonance images are exploited in (Bertelli et al.) where radiomics is combined with the adoption of artificial intelligence with the aim to provide an indicator about the aggressiveness of prostate cancer. A comparison between the radiomic features obtained from multiparametric and biparametric MRI is proposed in (Chen et al.), in this case with the aim of achieving an accurate prediction of the PI-RADS score. The prediction of the PI-RADS score is also proposed by the research paper (Li et al.), highlighting the fact that radiomics is becoming crucial for the detection of prostate lesions. Reliability is certainly one of the fundamental aspects of any methodology applied to such a critical area as medical diagnostics; this is the reason why authors in (Urraro et al.) investigate the state-of-the-art papers about reliability of radiomics. Another survey, related to metastatic prostate cancer detection is provided by the paper) (Kendrick et al.), while in the paper (Giannini et al.), artificial intelligence is once again exploited to build models starting from radiomic features, this time obtained from multi-parametric MRI.

It is our pleasure to present you our Editor’s Choices of the current Frontiers in Oncology issue.

## Author contributions

All authors listed have made a substantial, direct, and intellectual contribution to the work and approved it for publication.

## Conflict of interest

The authors declare that the research was conducted in the absence of any commercial or financial relationships that could be construed as a potential conflict of interest.

## Publisher’s note

All claims expressed in this article are solely those of the authors and do not necessarily represent those of their affiliated organizations, or those of the publisher, the editors and the reviewers. Any product that may be evaluated in this article, or claim that may be made by its manufacturer, is not guaranteed or endorsed by the publisher.

## Footnotes

1
**Cancer Facts & Figures 2022, American Cancer Society.**

